# DendroPrime as an adhesion barrier on fracture fixation plates: an experimental study in rabbits

**DOI:** 10.1177/1753193420932477

**Published:** 2020-06-19

**Authors:** Johanna von Kieseritzky, Henrik Alfort, Viktor Granskog, Daniel Hutchinson, Patrik Stenlund, Yalda Bogestål, Marianne Arner, Joakim Håkansson, Michael Malkoch

**Affiliations:** 1 Department of Clinical Science and Education and the Department of Hand Surgery, Karolinska Institutet, Stockholm, Sweden; 2 Department of Fibre and Polymer Technology, KTH Royal Institute of Technology, Stockholm, Sweden; 3 Biomedical Bonding AB, Täby, Sweden; 4 RISE Research Institutes of Sweden AB, Department of Biological function, Borås, Sweden; 5 Department of Laboratory Medicine, Gothenburg University, Gothenburg, Sweden

**Keywords:** Fracture, plate fixation, adhesion barrier, rabbit, flexor tendon

## Abstract

We tested the anti-adhesional effect of a new thiol-ene-based coating in a rabbit model. In 12 New Zealand white rabbits, the periosteum and cortex of the proximal phalanx of the second toe of both hind paws was scratched. Stainless steel plates were fixated with screws. One plate was coated with DendroPrime and the other left bare. The non-operated second toes of both hind paws of an additional four rabbits served as controls. Seven weeks after surgery, the soft tissue adhesion to the plates was evaluated macroscopically, and joint mobility was measured biomechanically. Toe joint mobility was about 20% greater and statistically significant in specimens with coated plates compared with the bare plates. Soft tissue overgrowth and, in some cases, synovitis or adhesions between the plate and the tendon were observed on all bare plates but not on any of the coated plates. We conclude that the thiol-ene-based coating can improve joint mobility by about 20%. This material has a potential to reduce adhesion around plates in fracture surgery.

## Introduction

Open reduction and plate fixation of phalangeal fractures often results in adhesion formation and finger stiffness (Brei-Thoma et al., 2015; Egloff et al., 2012; Kurzen et al., 2006; Onishi et al., 2015; Page and Stern, 1998; von Kieseritzky et al., 2017). The short distance between bone and tendons is further reduced by the osteosynthesis materials, and overgrowth of callus can also affect tendon gliding (Brei-Thoma et al., 2015). We have reported an incidence of reoperation of more than 40%, including hardware removal and tenolysis due to postoperative adhesions in phalangeal fractures (von Kieseritzky et al., 2017). The associated costs for the health care sector are substantial (de Putter et al., 2012).

Adhesion barriers have previously been tested in hand surgery; however, these studies have mostly focused on flexor tendon injury models (Golash et al., 2003; Hagberg and Gerdin, 1992; Hakansson et al., 2012; Lees et al., 2015; Wiig et al., 2011). Kappos et al. (2016) reported on the use of an adhesion barrier in fracture surgery using a cellulose membrane to cover phalangeal plates with short term positive effects.

Another problem with plate osteosynthesis in general in orthopaedic surgery is the overgrowth of soft tissue and bone on the plates, which makes hardware removal difficult (Hayes and Richards, 2010). Considering the number of hardware removals performed annually, facilitating the removal and shortening the operation time would be beneficial (Bostman and Pihlajamaki, 1996; Busam et al., 2006; Hanson et al., 2008).

High-performance thiol-ene composites have proven to be highly biocompatible without affecting bone healing in a related study exploring their use as a bone adhesive (Granskog et al., 2018). The aim of the present study was to evaluate DendroPrime, a novel thiol-ene-based coating as an adhesion barrier between a plate and a flexor tendon in an in vivo rabbit model. We hypothesized that the anti-adhesion properties of the coated metal plates would significantly improve the mobility of the distal interphalangeal (DIP) joint compared with bare metal plates.

## Materials and methods

### DendroPrime

The coating consists of a primer and an adhesive top layer, which are applied sequentially to the metal implant. Both components contain alkene and thiol monomers, which rapidly undergo a thiol-ene coupling reaction when exposed to high-energy visible light. The primer provides durable bonding to metal in wet and dry environments, while the adhesive top layer is a composite containing high levels of hydroxyapatite and demonstrates tissue-friendly and anti-adhesive properties. The coating can be applied directly during surgery and is ready in seconds using high-energy visible light.

### Animal model

Sixteen female New Zealand white rabbits (body weight approximately 2.5 kg; Envigo, Venray, The Netherlands) were used. The study was performed after approval by the local animal ethical committee. The animals were housed 2 weeks before surgery with free access to water, pellets (Lactamin AB, Kimstad, Sweden) and daily fruit or carrots.

The model in this study was similar to the model described by Olmarker et al. (2010). Anaesthesia was induced with medetomidin (0.28 mg/kg, Domitor Vet, Orion Pharma, Stockholm, Sweden) and ketamin (17 mg/ml, Ketaminolvet, Intervet, Stockholm, Sweden). A booster dose of ketamin (10 mg/kg) was administered 40 minutes after induction, when needed. After the surgery, atipamezol (1.5 mg/kg; Antisedan, Orion Pharma) was given subcutaneously. A single dose of 100 mg cefuroxime (Zinacef; GlaxoSmithKline, Mölndal, Sweden) was administered intravenously in an ear vein during surgery. A subcutaneous injection of buprenorphine (0.3 mg/kg; Temgesic, Schering-Plough, Brussels, Belgium) was given for pain relief during surgery and for 5 days postsurgery.

The surgeries were performed under sterile conditions on both hind paws of 12 rabbits. An additional four non-operated rabbits served as controls. The rabbits were shaved on the plantar side of the hind paws after anaesthesia was induced. A straight incision was made on the plantar aspect of the proximal phalanx of the second toe. The tendon sheath was opened, leaving the pulleys intact, and the flexor tendons were pulled to the side using vessel loops. A scratch was made with a scalpel blade in the plantar periosteum and cortex of the bone, and a two-hole Arbeitsgemeinschaft für Osteosynthesfragen (AO) 1.3 mm stainless steel plate was attached to the bone using 1.3 mm screws. DendroPrime was applied to the metal plate and screws on one of the paws of each rabbit by coating the implant with a layer of primer and a top-coat of the thiol-ene composite before curing with high-energy visible light. On the opposite paw, the metal plate was left uncoated. The surgeons were blinded to the treatment allocation until the moment of DendroPrime application. The tendons were allowed to glide over the plate and the coating, and the tendon sheath was closed using a running suture (PDS 5-0, Ethicon, Sollentuna, Sweden). The skin was closed with a running intracutaneous suture (Monocryl 5-0, Ethicon Inc. St-Stevens, Woluwe, Belgium). The rabbits were killed at the end of Week 7 after surgery, and the operated toe was dissected out with its attachment to the metatarsus intact.

### Biomechanical testing

**Figure 1.**
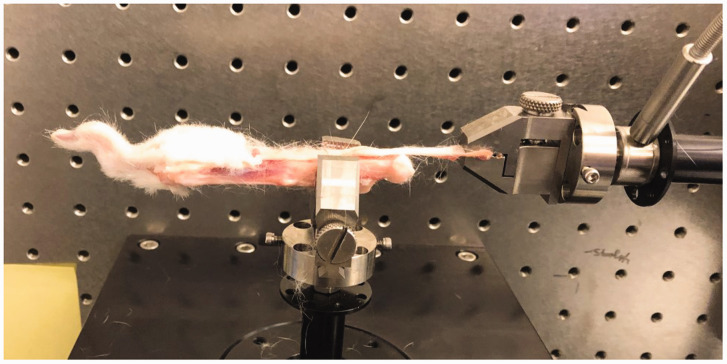
Biomechanical testing device. The paw was secured, and the flexor digitorum profundus tendon was attached to the tension testing device.

All operated and control digits were evaluated biomechanically and compared regarding mobility as previously described by Olmarker et al. (2010). The paw was secured, and the flexor digitorum profundus (FDP) tendon was attached to a tension testing Planar Biaxial TestBench Instrument (TA Instruments – ElectroForce System Group, Eden Prairie, MN, USA), pulling the tendon at a rate of 0.4 mm/s while simultaneously measuring the resulting forces by a 22.2 N load cell (Figure 1). The angles of the metatarsophalangeal, proximal interphalangeal and DIP joints in the digit were measured at pulling forces 0, 0.5, 1, 2, 3, 4 and 5 N using snapshots taken from the film sequence recorded during testing. Image J software (version 1.52i) was used for angle measurements. All angles were measured, blinded, and controlled repeatedly by author one and two. The joint angles were normalized against the angles at 0 N force, giving 0° of flexion at 0 N.

### Visual inspection

During dissection, all specimens were thoroughly inspected, and the presence of adhesions, synovitis and signs of coating degradation was documented. Blinding of the observer was not possible since the presence of the coating was obvious. No attempt was made to quantify the amount of synovitis, adhesion or degradation.

### Statistical analysis

The statistical method used for comparison of toes with bare plates and coated plates was the Wilcoxon rank sign test. The statistical method used to compare native non-operated toes with toes with bare plates was one-way analysis of variance with Tukey HSD as post hoc test. *P* ≤ 0.05 was considered significant.

## Results

One rabbit was excluded from the study due to a technical failure. Another rabbit was re-sutured on one toe on the third postoperative day as it had removed the sutures by licking them. However, the soft tissues, tendon and implant were not affected and after re-suturing the wound healed without complications.

### Biomechanical testing

**Figure 2.**
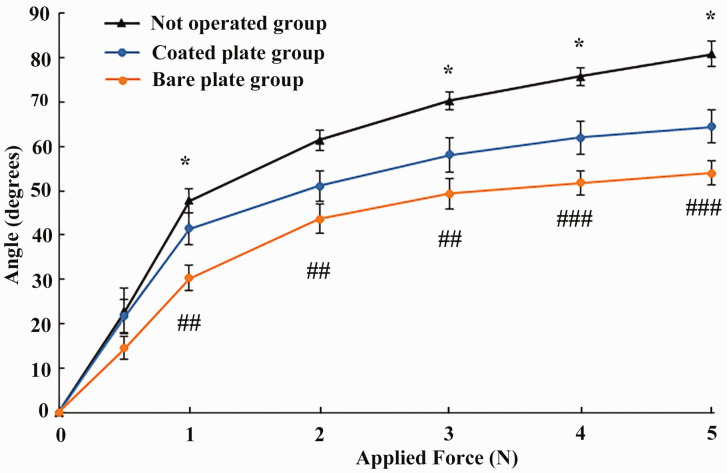
The measurements of distal interphalangeal joint angle at the pulling forces of 0.5, 1, 2, 3, 4 and 5 N. The points in the graph represent mean and the error bars show SEM. ^#^Statistically significant differences between native and operated digits with bare plates. *Statistically significant differences between operated digits with bare plates (*n* = 11) and plates with DendroPrime (*n* = 11). *p < 0.05; ^##^*p* < 0.01; ^###^*p* < 0.001.

The measurements of the toes with bare plates had statistically significantly reduced DIP joint mobility compared with the non-operated native toes in all measuring points except at 0.5 N (*p* < 0.05 or *p* < 0.01) (Figure 2). Coating of the plate significantly increased the mobility in the rabbit toes DIP joints compared with bare plates at the measuring points 1, 3, 4 and 5 N (*p* < 0.05 or *p* < 0.01) (Figure 2). There was approximately a 20% increase in mobility in specimens with coated plates compared with the bare plate controls with 1–5 N load. Compared with non-operated control samples, the toe joint mobility in both operated groups was significantly less (*p* < 0.05 or *p* < 0.01), as shown in Figure 2.

### Visual inspection

**Figure 3.**
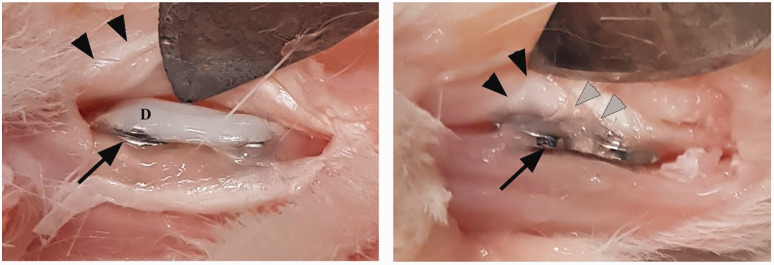
DendroPrime-coated plate and screws without any soft tissue adhesions (left). Bare plate covered with soft tissue adhesions between the tendon and the plate (right). Arrows indicate plate, black arrow heads tendon and grey arrow heads soft tissue adhesions. D: DendroPrime.

In the specimens with bare plates, overgrowth of soft tissue on the implant and, in some cases, synovitis around the plate and the tendon or adhesions between the plate and the tendon was observed (Figure 3). On the coated plates there was no soft tissue overgrowth and no synovitis. The coating itself showed no signs of degradation, and there was no surrounding inflammation.

**Figure 4.**
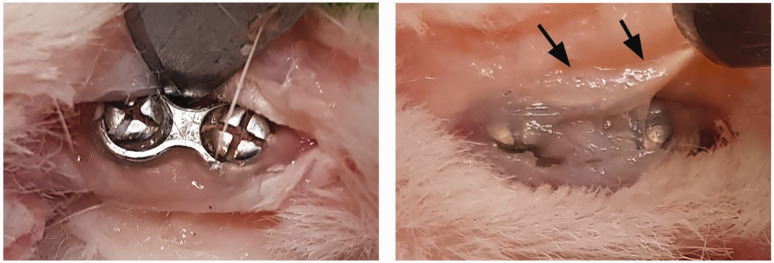
Plate after removal of DendroPrime, showing clean screws (left). Bare plate and screws covered with synovitis, indicated by arrows (right).

Synovitis covering the screws on the bare plates made removal of the plates difficult (Figure 4). Removal was considered less complicated on the toes with the coated implants. The thiol-ene composites were easily removed from the plate using a scalpel blade, and the plate could be extracted without effort.

## Discussion

Soft tissue adhesions can occur both against implants, bone and periosteum. Our group has previously demonstrated a good compatibility with surrounding soft tissue of thiol-ene composites while investigating its use in a fibre-reinforced adhesive patch for fracture fixation (Granskog et al., 2018). The present study focused on adhesions to plates. The application of DendroPrime onto the implanted plates demonstrated statistically significantly better mobility in the DIP joint compared with the uncoated, bare metal plates. Furthermore, the coating prevented the growth of soft tissue around the implant, which made removal easier compared with uncoated plates. We hypothesise that the preventive action of DendroPrime on soft tissue adhesions could reduce the need for plate removal after successful fracture healing.

A study in hand surgery related to the reduction of adhesions after fracture surgery was published by Kappos et al. (2016). The anti-adhesional effect of a methyl cellulose barrier seen at 6 weeks postoperatively had disappeared at 6 months due to degradation of the product. Conversely, DendroPrime showed no sign of degradation or resorption, such as inflammation, change of texture or size of the coating at 7 weeks.

**Figure 5.**
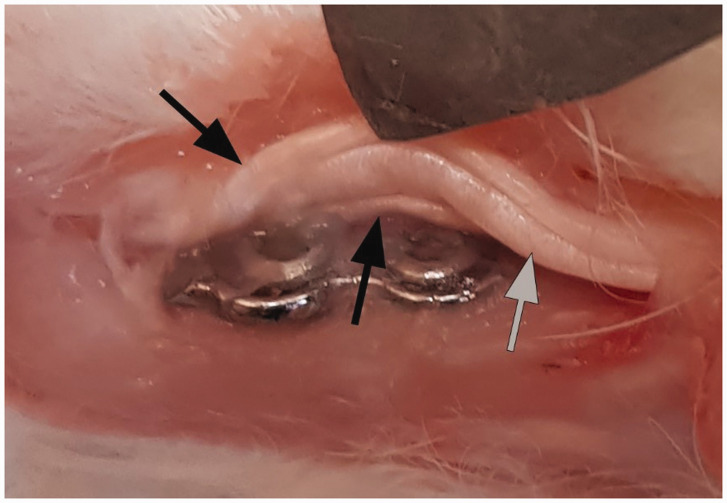
Anatomy of the tendons resulting in a limited contact area between flexor digitorum profundus tendon and plate. Black arrows indicate the flexor digitorum superficialis tendon and grey arrow the flexor digitorum profundus tendon.

In the present study, the differences in DIP joint mobility between the two treatment groups was limited. The measurement of the DIP joint angles was the primary outcome since the FDP tendon, gliding over the implant, is the only tendon affecting this joint on the flexor side. The limited differences in DIP joint mobility between the toes with coating and with bare plates were likely due to the flexor digitorum superficialis tendon, which partially limits the contact area of the FDP tendon to the implants (Figure 5). The rabbits' large range of motion and fast recovery may also play a role.

The rabbit model used in this study was originally designed for analysing effects on adhesions after flexor tendon repair. To be applicable in this study, the model was modified by simulating a phalangeal fracture and attaching a plate on the phalanx. A limitation of the model was the short phalanges of the rabbit toes, which restricted the length of the implant and thus the limited contact area between the FDP tendon and the plate (Figure 5). The periosteum and cortex of the bone was scratched before application of the plate to induce an injury and a repair response in the bone. It was not possible to make a reproducible complete fracture due the limited space on the small phalanx. Blinded visual inspection of the synovitis and adhesions formation was not possible since the coating was clearly visible. However, the presence or lack of adhesions and synovitis was obvious in each case. The findings of our study are based on an experimental rabbit model and should not be extrapolated to fractures in humans without further studies.
